# Genomic selection in a kiwiberry breeding programme: integrating intra- and inter-specific crossing

**DOI:** 10.1007/s11032-025-01550-8

**Published:** 2025-03-07

**Authors:** Daniel Mertten, Catherine M. McKenzie, Samantha Baldwin, Susan Thomson, Edwige J. F. Souleyre, Michael Lenhard, Paul M. Datson

**Affiliations:** 1 The New Zealand Institute for Plant and Food Research Ltd, Auckland, 1142 New Zealand; 2 Institute for Biochemistry and Biology, University of Potsdam, 14476 Potsdam-Golm, Germany; 3 The New Zealand Institute for Plant and Food Research Ltd, Te Puke, 3182 New Zealand; 4 The New Zealand Institute for Plant and Food Research Ltd, Lincoln, 7608 New Zealand; 5 Kiwifruit Breeding Centre, Auckland, 1142 New Zealand

**Keywords:** Autotetraploidy, Genomic estimated breeding value, Predictive ability, *Actinidia melanandra*, *A. arguta*-complex

## Abstract

Inter-specific hybridisation between natural populations within the genus *Actinidia* is a common phenomenon and has been used in breeding programmes. Hybridisation between species increases the diversity of breeding populations, incorporating new desirable traits into potential cultivars. We explored genomic prediction in *Actinidia* breeding, focusing on the closely related species *Actinidia arguta* and *Actinidia melanandra*. We investigated the potential of genomic selection by analysing four quantitative traits across intra-specific *A. arguta* crosses and inter-specific crosses between *A. arguta* and *A. melanandra*. The continuous distributions of the studied traits in both intra-specific and inter-specific crosses indicated a polygenic background. A linear mixed model approach was used, incorporating the factor of year of season and a marker-based relationship matrix instead of pedigree as a random effect. After evaluation, the best model was applied to assess variance components and heritability for each quantitative trait. Expanding beyond intra-specific crosses, predictive ability was calculated to investigate inter-specific cross effect. Considering predictive ability, this study explored the impacts of sample size and population structure. A reduction in sample size correlated with decreased predictive ability, while the influence of population structure was particularly pronounced in inter-specific crosses. Finally, the prediction accuracy of genomic estimated breeding values, for parental genotypes, revealed an inter-species effect on prediction confidence. Considering the imbalance in genotype numbers between intra- and inter-specific cross populations, this research highlights the difficulty of genomic prediction in hybrid populations. Understanding prediction accuracy in inter-species crossing designs provides valuable insights for optimising genomic selection.

**Supplementary Information:**

The online version contains supplementary material available at 10.1007/s11032-025-01550-8.

## Introduction

Plant breeding has long been a cornerstone of agricultural advancement, and genomic selection has the potential to revolutionise crop improvement. Modern plant breeding has incorporated statistical analysis tools and genomic information, to enhance parental selection ‒ a practice originally derived from animal breeding.

Genetic, environmental, and additional influences, such as crop management, contribute to the variability in any trait. To effectively select for a trait, a significant portion of the variation should arise from heritable genetic factors that segregate, rather than being predominantly influenced by environmental factors. Statistical methods, like Best Linear Unbiased Predictions (BLUPs), have been developed in animal breeding and adopted in plant breeding to assess genetic effects by estimating variance components and predicting breeding values (Patterson and Thompson 1971; Henderson 1974, Piepho et al. 2008).

Considering phenotypic information of relatives via pedigree has assisted animal and plant breeding. When sex-linked traits can only be observed in one sex, the opposite sex requires progeny testing to estimate breeding values. Incorporating marker-based relationship information between individuals within a breeding population has overcome the need for additional progeny testing and improved the reliability of breeding value prediction, especially when no phenotypic information is available (as in sex-linked traits) (VanRaden 2008; de Bem Oliveira et al. 2019; Mertten et al. 2023).

The genus *Actinidia* encompasses more than 50 species with 75 taxa (Li et al. 2007a, b). All species, described as dioecious, grow as long-lived, perennial, woody vines across their native habitat from Siberia through East Asia, to Southeast Asia and across to India. Fruits of the *Actinidia* genus are universally berries. Morphological studies have classified all *Actinidia* species into two groups: a) smooth-skinned fruiting (SSF) and hairy-skinned fruiting (HSF) species (Huang 2014). Currently, only two species within the HSF group have been widely commercialised: *Actinidia chinensis*, which includes two subspecies (*A. chinensis* Planch. and *A. chinensis* var. *deliciosa* (A. Chev.) A. Chev.), and *Actinidia eriantha* Benth (Huang and Ferguson 2007; Li et al. 2007a, b, c; Liao et al. 2022). Another promising species within the *Actinidia* genus is *Actinidia arguta* (Sieb. and Zucc.) Planch. ex Miq., part of the *A. arguta*-Complex within the SSF group, also known as kiwiberries or hardy berries. This species exhibits small fruits with predominantly green flesh and edible skin, along with strong tolerance to winter cold. A more distantly related species, *Actinidia melanandra* Franch., unique to China, is found within the same complex. Within *A. arguta*, many genotypes have been identified with diverse fruit colour ranging from green to purple, whereas *A. melanandra* bears fruit generally reported to have red to purple flesh, and skin colour differs in intensity; in the accessions of this study, the fruit also displayed this red to purple range (Kataoka et al. 2010; Asakura and Hoshino 2016; Zhang et al. 2017).

In many plant species, the estimation of breeding values is confounded by polyploidy due to whole-genome duplication, common in angiosperms, playing a crucial role in the adaptation to environmental conditions (Soltis et al. 2004, 2015; Comai 2005; Baduel et al. 2018). Approximately 15% of angiosperm speciation events have been linked to polyploid occurrences, as estimated by Wood et al. in 2009. Various types of polyploids are characterised by the number of coexisting chromosome sets and the pattern of chromosome inheritance during meiosis. The two extreme forms are auto- and allopolyploidy, while a mixed form, known as allo-autopolyploidy, is also present. Autopolyploids arise from genome duplication or the fusion of two closely related species, exhibiting non-preferential chromosome pairing during meiosis. In contrast, allopolyploids result from the combination of chromosome sets from two or more distantly related species and demonstrate preferential chromosome pairing behaviour during meiosis (Sears 1976; Soltis and Soltis 1999; Soltis et al. 2007). The genus *Actinidia* presents a range of ploidy levels, spanning from diploid to octoploid and occasionally higher. These ploidy levels vary within and between species. Studies on genome size have revealed a consistent basic chromosome number of *x* = 29 across all *Actinidia* species (Watanabe et al. 1990; Huang and Ferguson 2007).

*A. arguta* exemplifies a diverse ploidy range and its native occurrence. Specifically, diploid *A. arguta* populations are more prevalent at lower altitudes, while hexaploid populations are found at higher altitudes with colder winters (Kataoka et al. 2010). Tetraploid *A. arguta* populations are both more common and widely distributed, likely conferring a selective advantage in adapting to different environmental conditions. Other *Actinidia* species also demonstrate a similar correlation between ploidy range, altitude, and geographic distribution (Kataoka et al. 2010; Li et al. 2010; Zhang et al. 2017). A recent study described tetraploid *A. arguta* as an autopolyploid, originating from a whole-genome duplication event in diploid *A. arguta* (Zhang et al. 2024).

Compared with currently cultivated commercial kiwifruit species, kiwiberries appear to hold the most promise in breeding programmes (Williams et al. 2003). The existing breeding strategy for kiwifruit species, such as *A. arguta*, mirrors techniques utilised in animal breeding. This method involves selecting genotypes through processes like single-seed descent while also utilising pedigree records to maintain crucial relationship data. Due to the sex linkage of fruit traits in dioecious crop breeding, only female plants bear fruit. Consequently, female genotypes displaying desirable traits are carefully chosen and clonally propagated for subsequent commercial cultivation.

Selecting superior individuals becomes challenging when phenotype observations are not feasible, such as in the case of male vines within a cross. When pedigree information is available, the breeding values of fruit characteristics in male genotypes within the same cross are estimated as family means and cannot be distinguished individually. Therefore, selecting male parents requires progeny testing since they provide no phenotypic information on their genetic background for the breeder. This process is time-consuming and expensive, as kiwifruit vines need an establishing period of around 3 years before flowering.

Hence, there is a necessity to develop methods allowing the individual estimation of trait values for non-expressed traits of *Actinidia* species. Recently, genomic methods have been developed to enable this prediction (Testolin 2011; Datson et al. 2017; Cheng et al. 2019). In polyploids with their multiple homologous chromosome sets, allele dosage information is crucial for estimating marker-based additive variance–covariance relationships between individuals to predict breeding values. To date, there are only a few publications addressing the application of genomically estimated breeding values (GEBV) to the breeding of autotetraploid kiwiberries (Mertten et al. 2023; Zhang et al. 2024).

Within the genus *Actinidia*, the variability of inter-species hybridisations is a common phenomenon, particularly in habitats where varieties and species overlap. Introgression crossing within *Actinidia* germplasms offers a powerful method for improving new cultivars by incorporating desirable traits from different species (Huang and Liu 2014). However, inter-specific hybridisations must overcome crossing barriers due to ploidy and morphological differences. In *Actinidia*, crosses between species at the same level of ploidy are more successful than crosses carried out using species with unbalanced chromosome numbers in the parents. Additionally, a previous study showed that inter-ploidy crosses tended to be more successful when the female parent had a lower ploidy level than the male parent (Pringle 1986; Hirsch et al. 2001; Asakura and Hoshino 2018). Morphological differences in flower organs tend to be a barrier to inter-specific crosses due to the significant variation in the size of flowers between species (Pringle 1986).

In this study, we investigated the ability to predict GEBVs in an *A. arguta* breeding population. To account for the potential influence of inter-species crosses, we extended our analysis to include two distantly related *Actinidia* species within the *A. arguta* complex. We examined the fixed-effect structure of linear mixed models to enhance predictive ability in inter-species breeding populations, along with the effects of population structure, training population size, and their impact on prediction accuracy. A reduction in sample size moderately affected the breeding value accuracy of three male parents, with a strong relationship effect observed between these males and the training set. Our findings lay the foundation for integrating genomic selection into inter-species breeding for kiwiberry improvement.

## Material and methods

### Plant population and population structure

The parental breeding programme at The New Zealand Institute for Plant and Food Research Limited (PFR) included a seedling population resulting from the crossbreeding of tetraploid *A. arguta* × *A. arguta* and *A. arguta* × *A. melanandra*. This seedling population comprises two incomplete factorial crossing designs, encompassing both intra- and inter-species crosses. The first incomplete crossing design comprised two females crossed with 13 males (2 × 13), while the second factorial design included 13 females crossed with three males (13 × 3), as detailed in Supplementary Table 1. From the 13 × 3 factorial design, an additional balanced subset of 7 × 3 crosses were selected, ensuring that all female parents were successfully crossed with the three male parents (Supplementary Table 2).

In 2014, at the PFR Motueka Research Centre, 1832 seedlings from 48 intra- and seven inter-species crosses were planted. For each cross, a minimum of 20 randomly selected seedlings, including both male and female plants, were grouped in sets of seven for planting in the field trial. Clonal replicates were not included in the trial. The number of offspring per cross ranged from a minimum of 2 to a maximum of 80 seedlings. On average, there were 33.3 seedlings per cross, with a median of 38.0.

The spatial arrangement involved planting with a spacing of 0.5 m within rows, while the distance between rows extended to 3.0 m. The selected cultivation method was the pergola support system, commonly employed in New Zealand's production practices. These plants were allowed to establish in the field for two years before the first fruit and vine assessments. After undergoing winter pruning, two canes from the current growing season were horizontally trained during the summer and kept for future phenotypic observations (Mertten et al. 2023).

### Phenotyping and trait analysis

Throughout this research, we examined one vine trait (fruit load) and the three fruit traits: fruit weight, dry matter percentage and ripe soluble solids content. Within the five-year trial, fruit load was recorded from 2017 and 2018 and scored from 0 to 9, representing fruit load intensity (Supplementary Table 3). However, individuals with a score of zero were excluded from the study as this indicated the absence of fruits (Mertten et al. 2023).

Regarding fruit traits, fruit weight and dry matter percentage were assessed from 2017 to 2019, at harvest stage, when fruit maturity was indicated by > 90% of seeds being black. Only firm fruits were harvested (Beatson et al. 2009). Fruit weight was recorded as the average weight (in grams) of 30 randomly chosen fruits from each vine. Three fruits were selected randomly to measure average dry matter percentage; cross-sectional slices measuring 2–5 mm were taken following the methodology proposed by Fenton and Kennedy in 1998 (Beatson et al. 2009; Mertten et al. 2023).

For the assessment of ripe soluble solids content, ten fruits were sampled at harvest stage and stored at 4 °C for 28 days, followed by a final day at room temperature to facilitate ripening. Ripe soluble solids content was measured as an average of three of these ripe sampled fruits using a digital pocket refractometer (ATAGO®) in both 2018 and 2019 (Beatson et al. 2009).

We employed the "moments" R-package v. 0.14.1 to analyse the distribution of fruit load, fruit weight, dry matter percentage and ripe soluble solids content (Komsta and Novomestky 2022; Mertten et al. 2023; R Core Team 2023).

### Genotyping and principal component analysis

DNA was isolated from young leaf tissue by Slipstream Automation (Palmerston North, New Zealand) and the concentration of double-stranded DNA was standardised to 500 ng per sample for the high-throughput targeted multiplex amplicon-sequencing platform Flex-Seq® Ex-L of RAPiD Genomics (RAPiD Genomics Gainesville, FL, USA). The selection process for target SNPs using the Flex-Seq® Ex-L platform (RAPiD Genomics, Gainesville, FL, USA) prioritised heterozygosity and Hardy–Weinberg equilibrium, excluding multi-mapping probes, resulting in 3,300 exonic targets distributed across the gene space. Paired-end sequencing using the 2 × 150 Illumina NovaSeq platform ensured a sequence coverage of > 100x, generating 250–350 bp fragments for high-resolution analysis (Clare et al. 2024). The resulting sequence reads were aligned to the diploid male reference genome *A. chinensis* var. *chinensis* ‘Russell’ by employing BWA-MEM software and SAMtools (Li 2013; Danecek et al. 2021; Tahir et al. 2022; Mertten et al. 2023) with default settings. ANGSD was used for SNP calling with region selection based on target intervals (Korneliussen et al. 2014). Dosage estimation of tetraploid *A. arguta* × *A. arguta* population and SNP filtering were performed using the R-package "Updog" V2, considering allele bias (0.5 < bias < 2), over-dispersion (od < 0.02), and sequencing error (seq < 0.01) (Tahir et al. 2020; Mertten et al. 2023; R Core Team 2023). Dosage genotypes were called using an empirical Bayesian approach, assuming tetraploid (4*x*) as 0 (AAAA), 1 (AAAB), 2 (AABB), 3 (ABBB), and 4 (BBBB) (Gerard et al. 2018; Mertten et al. 2023).

The population structure of the two factorial crossing populations was investigated after filtering genotype quality using dosage calls. Principal component analysis (PCA) was performed using the “prcomp” function from the “stats” v. 4.3.0 R-package (R Core Team 2023).

### Linear mixed model and genetic analysis

Genomic Estimated Breeding Values of four quantitative breeding traits were calculated using the function “kin.blup” from the R-package “rrBLUP” v. 4.6.2 and “asreml” from the package “ASReml-R” v. 4.1.0.149 (Endelman 2011; Gilmour et al. 2015; Butler 2021; R Core Team 2023). A univariate linear mixed model was applied to calculate BLUPs, treating genotypic effects as random and incorporating the overall population mean along with additional factors (year of season, cross-type, year‒cross-type interaction).

The following genomic selection model equation was applied:

$$y=\mu +Xb+Za+e,$$

where $y$ represents a column vector of phenotypic values for the trait under analysis, $\mu$ stands for the overall population mean, $b$ is a vector associated with fixed effects with the incidence matrix $X$. Additionally, $a$ denotes the unobserved random effect related to genotypes, with $a\sim \text{N}(0,\mathbf{G}{\sigma }_{\text{a}}^{2})$, where ${\sigma }_{\text{a}}^{2}$ is the additive variance and $Z$ the incidence matrix of genotypes and $e$ is the random residual effect with $e\sim \text{N}(0,\mathbf{I}{\sigma }_{\text{e}}^{2})$, where ${\sigma }_{\text{e}}^{2}$ represents the residual variance.

The marker-based relationship matrices for two factorial crossing designs, involving inter- and intra-species crosses of *A. arguta* and *A. melanandra*, was computed using the R-package “AGHmatrix” v. 2.0.4 (Amadeu et al. 2016; R Core Team 2023). In this study on autotetraploids, a total of 7259 markers were used to construct the realised relationship matrix, following the methods described by VanRaden (2008) and Ashraf et al. (2016). Variance components and residuals were estimated using the Restricted Maximum Likelihood (REML) methodology provided by the R-package “rrBLUP” and “ASReml-R”. The narrow-sense heritability (${h}_{\text{NS}}^{2}$) across multiple years of observations on an individual plant basis was calculated as the proportion of additive variance component ${\sigma }_{\text{a}}^{2}$ to the total phenotypic variance component ${\sigma }_{\text{p}}^{2}$, as described by Falconer and Mackay (1996): ${h}_{\text{NS}}^{2}=\frac{{\sigma }_{\text{a}}^{2}}{{\sigma }_{\text{p}}^{2}}$.

The determination of BLUP estimation accuracy we followed Henderson's 1975 definition:

$$Accuracy= \surd (1-\frac{PEV}{{\sigma }_{a}^{2}{G}_{ii}})$$

Here, *PEV* is the predicted error variance of breeding values for each individual, ${\sigma }_{a}^{2}$ is the additive variance, and ${G}_{ii}$ is the diagonal element of the variance–covariance matrix with ${G}_{ii}$ = 1 + *F*, where *F* is the inbreeding coefficient of individual *i* (Henderson 1975; Mrode and Thompson 2014; Gilmour et al. 2015; Isik et al. 2017).

### Prediction accuracy and leave-one-out cross-validation

**Fig. 1:**
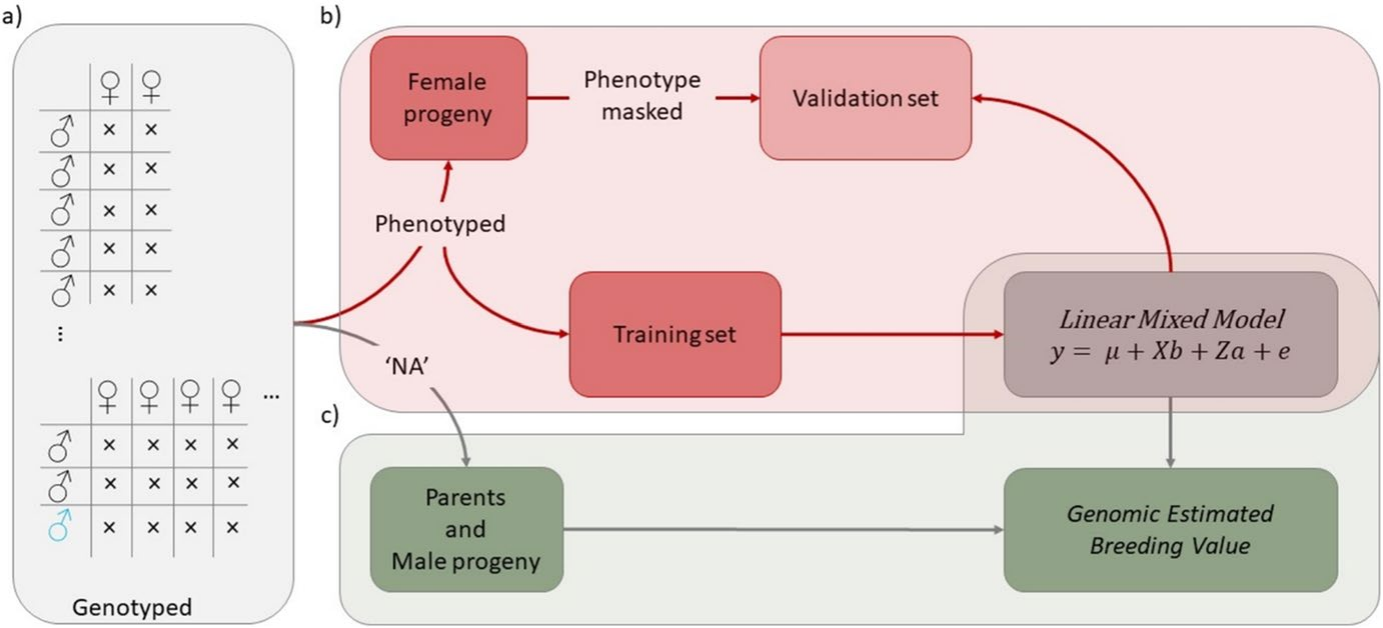
Prediction of Genomic Estimated Breeding Values for female-related traits in intra- and inter-species crosses. a) The breeding population consisted of two factorial crossing designs: *Actinidia arguta* × *Actinidia arguta* (black) and *Actinidia arguta* × *Actinidia melanandra* (blue). Genotyping was performed for parental genotypes, female and male progeny. b‒c) The population was then divided into the female progeny with phenotype records and individuals with no observations available (indicated by ‘NA’), which included mainly male progeny and parental genotypes. b) The female progeny population was further subdivided into a training set for training the linear mixed model and a validation set for assessing predictive accuracy through observation masking. c) Genomic Estimated Breeding Values for female and male progeny, as well as for parental genotypes, were calculated, taking into account the linear mixed model and phenotype observations

In this study, 15 female parents, 16 male parents, and 1832 progeny from the two factorial crossing population were genotyped (Fig. 1a). Female progeny were phenotyped and subsequently divided into a training set and a validation set (Fig. 1b).

Genotypic and phenotypic information were included for each individual within the training set. Parental and male genotypes were linked to the training set using the marker-based relationship matrix, and GEBV were computed (Fig. 1c). The accuracy of the model prediction was evaluated using leave-one-out cross-validation (LOO-CV), meaning each phenotyped female was counted once, either as a training set or a validation line with a masked observation. The prediction accuracy of the linear mixed model equation was validated using the correlation between predicted GEBV and mean observation. In addition, an analysis was performed on the impact of inter- and intra-specific crosses when relying solely on phenotypic information within sub-population AA × AA or sub-population AA × ME. The AA × AA sub-population comprised all intra-specific crosses of *A. arguta* of both factorials. Furthermore, an exploration was undertaken to understand the influence of different level of fixed effects (year, cross type, year‒cross type interaction) on model predictions.

### Principal component- and statistical analysis

Within each factorial crossing design, population structure was investigated using PCA of the marker dosage matrix, via the function “prcomp” from the R-package “stats” v. 4.3.0 (R Core Team 2023). For each factorial sub-population the common parental genotype was used to distinguish population structure.

The normality of trait distribution was assessed by calculating Pearson’s coefficient of skewness using the R-package “moments” v. 0.14.1 (Komsta and Novomestky 2022; R Core Team 2023). A negative skewness indicates a left-skewed distribution (left-tailed), while a positive skewness indicates a right-skewed distribution (right-tailed). A skewness coefficient between −0.5 and 0.5 suggests a normal distribution, between −1.0 to −0.5 or 0.5 to 1.0 indicates a moderately skewed distribution, whereas a skewness coefficient below −1.0 or above 1.0 indicates a highly skewed distribution (Bulmer 1979).

A variance analysis was conducted to investigate the equality of variances among the intra-species crosses of *A. arguta* and the inter-species crosses of *A. arguta* and *A. melanandra* sub-populations focusing on four breeding traits. Levene's test, was used, chosen for its suitability for distributions with moderate skewness and unbalanced population size. The Levene's test was carried out using the R-package “car” v. 3.0–10 (Fox and Weisberg 2019; R Core Team 2023).

A linear mixed model was utilised to predict breeding values and various independent variables, with the fixed effects of year (season), cross type (AA × AA, AA × ME), along with the two-way interaction. To assess the significance of each independent variable in the linear mixed model, a Wald Chi-squared Test was conducted using the “wald.asreml” function from the “ASReml-R” R-package (Gilmour et al. 2015; Butler 2021; R Core Team 2023). The correlation coefficient (predictive ability) and significance test for different models were calculated using the function “cor.test” from the R-package “stats” (R Core Team 2023).

Plots for visualisation were created using the R-packages “ggplot2” v. 3.4.2 and “patchwork” v. 1.1.1 (Wickham 2016; Pedersen 2020; R Core Team 2023).

## Results

Four quantitative traits were assessed: scored fruit load (0.5‒9), average fruit weight (in grams), average dry matter percentage, and ripe soluble solids content (in °Brix), in *Actinidia* crosses over two to three years. These traits showed continuous distributions across the total seedling population, which included both intra-specific (*A. arguta* × *A. arguta*) and inter-specific (*A. arguta* × *A. melanandra*) crosses. The ranges were as follows: fruit load from 0.5 to 9.0, fruit weight from 1.0 to 17.3 g, dry matter percentage from 12.0 to 29.3%, and soluble solids content from 9.1 to 22.4°Brix. All traits exhibited fairly to moderately right-skewness (i.e., positive skewness values) across multiple years (Supplementary Table 4).

Intra- and inter-specific populations were statistically compared using a total population that included both factorial designs, as well as a subset of the 13 × 3 factorial, consisting of seven female and three male parents (7 × 3 factorial). The intra-specific population showed lower skewness coefficients for all traits than the inter-specific population. The largest discrepancy in the skewness coefficient between intra- and inter-species crosses was observed particularly for dry matter percentage and soluble solids content, reflecting the smaller number of phenotyped female progeny in the AA × ME cross. Levene's test indicated no significant differences (*p*-value > 0.05) in variance between the intra- and inter-specific populations in the total population, suggesting homogeneity of variance. However, within the 7 × 3 factorial subset, a significant difference (*p*-value = 0.003) was found in average fruit weight, implying heterogeneity of variance between the populations (Table 1).

**Table 1:** Assessment of quantitative traits in intra- and inter-species *Actinidia* crosses. Four quantitative traits in intra- species (*Actinidia arguta* × *Actinidia arguta*) and inter-species (*Actinidia arguta* × *Actinidia melanandra*) crosses are examined. These traits include scored fruit load (0.5‒9), average fruit weight (in grams), average dry matter percentage, and average ripe soluble solids content (in °Brix). Key statistics such as sample size (N), mean, median, and skewness for each trait are shown for both cross types. Additionally, Levene’s test results, including *F*-value and *p*-value, are provided to evaluate the equality of variances between the intra- and inter-species crosses for each trait. The analyses involved a total population using a 2 × 13 and a 13 × 3 factorial design, with a separate analysis conducted on a subset of the 13 × 3 factorial, resulting in a 7 × 3 factorial

	AA×ME				Total population						7×3 factorial					
Trait					AA×AA				Levene’s test		AA×AA				Levene’s test	
	N	Mean	Median	Skew	N	Mean	Median	Skew	*F*-value	*p*-value	N	Mean	Median	Skew	*F*-value	*p*-value
Fruit Load (0.5‒9)	44	4.1	4.0	0.20	794	4.4	5.0	0.06	2.159	0.142	239	4.5	5.0	0.01	1.217	0.271
Fruit Weight (g)	43	6.1	5.9	0.80	789	8.0	7.7	0.57	0.742	0.389	235	7.1	6.9	0.56	9.072	0.003
Dry Matter (%)	43	17.6	17.2	0.70	782	20.7	20.6	0.12	0.337	0.562	234	20.8	20.7	0.09	0.276	0.599
Soluble Solids Content (°Brix)	40	13.8	13.5	0.60	769	16.1	15.9	0.19	0.274	0.601	231	16.2	16.0	0.15	0.655	0.419

### Population study

**Fig. 2:**
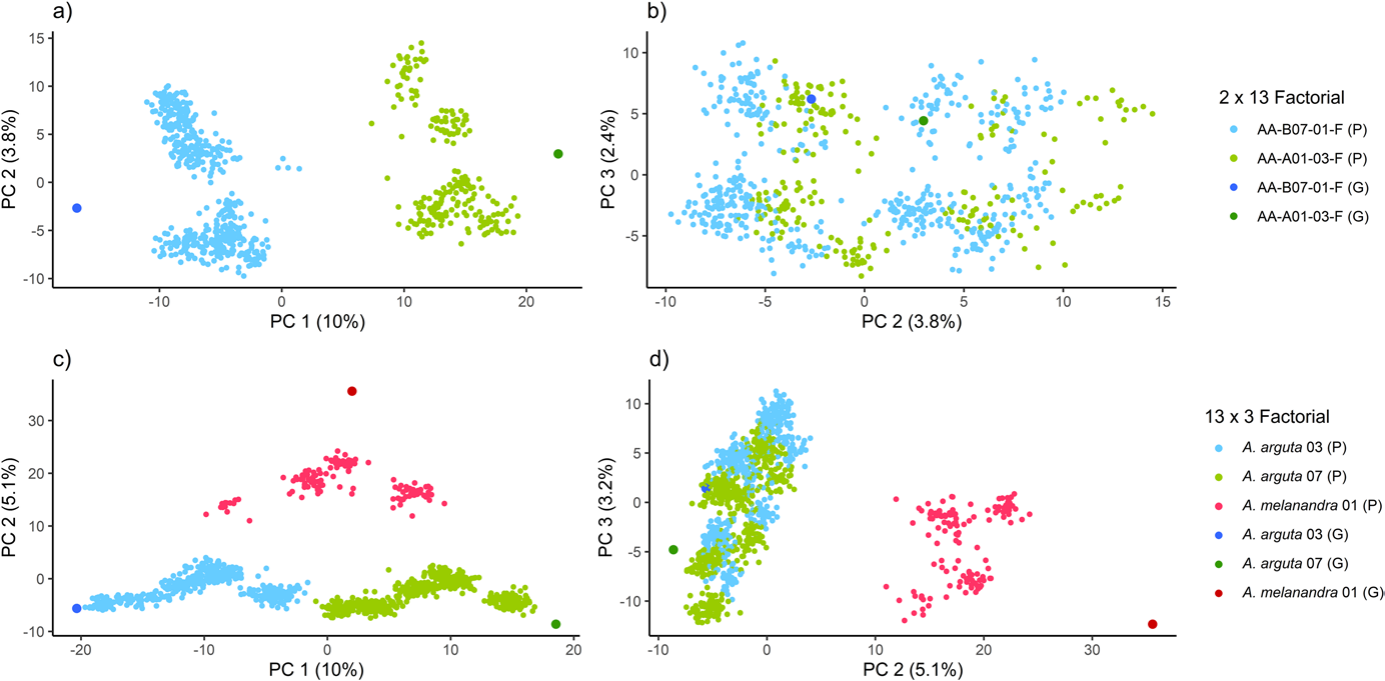
Genetic variance explained by principal components and population structure investigation. Principal component analysis explains the genetic variation in the *Actinidia arguta* × *Actinidia arguta* and *Actinidia arguta* × *Actinidia melanandra* crossing population in terms of percentage of explained variances. The correlation between PC1 and PC2, as well as between PC2 and PC3 is shown for 2 × 13 (a‒b) and 13 × 3 factorial (c‒d). Progeny genotypes (‘P’) are grouped according to their corresponding parents, and 'G' indicates parental genotypes

The current *Actinidia* breeding population comprises two species of the kiwiberry complex, *A. arguta* and *A. melanandra*. A total of 1863 individuals (seedlings and parental genotypes) were genotyped, and allele dosages were estimated. When the population structure was studied using PCA, common parents for the 2 × 13 factorial and 13 × 3 factorial were considered (Fig. 2).

In the 2 × 13 factorial, higher similarity of progeny genotypes due to their common female parents was observed, as revealed by first principal component (PC1). However, no further distinction within intra-species crosses was observed using PC2 and PC3 (Fig. 2a‒b).

In the 13 × 3 factorial crossing design, consisting of inter- and intra-specific crosses between *A. arguta* and *A. melanandra*, the PC1 distinctively grouped genotypes into inter-species crosses with their common male parents, *A. arguta* 03 and 07, shown in blue and green, respectively. Similarities between progeny genotypes and their common male parents were observed (Fig. 2c). The inter-species crosses, characterised by a common *A. melanandra* male parent (shown in red), were distinctly separated from the intra-species *A. arguta* crosses, as indicated by PC2. This finding suggests dissimilarities between crosses descended from the *A. melanandra* male parent compared with intra-species crosses (Fig. 2c). No further grouping within the 13 × 3 factorial design was observed when considering PC3 (Fig. 2d).

### Linear mixed model and fixed effect- and population structure

The analysis utilised a linear mixed model with a genetic marker-based relationship matrix to examine the impact of the factors of year and cross type, and the two-way interaction on four quantitative traits in *Actinidia* crosses. A Wald Chi-squared test was employed to determine the significance of each factor for traits such as fruit load, average fruit weight, dry matter percentage, and soluble solids content. The results showed that the year effect had a significant impact on all traits, indicating its consistent influence across the dataset. Cross type had a significant effect on average fruit weight, dry matter, and soluble solids content, while the interaction between year and cross type was only significant for average fruit weight, suggesting that these factors interact meaningfully for this trait alone (Table 2).

**Table 2:** Wald Chi-squared test results for fixed effects in linear mixed models of *Actinidia* cross population. Wald Chi-squared test results for fixed effects in linear mixed models applied to four quantitative traits are shown for scored fruit load (0.5‒9), average fruit weight (g), average dry matter (%), and average ripe soluble solids content (°Brix), in an *Actinidia arguta* × *Actinidia arguta* and *Actinidia arguta* × *Actinidia melanandra* cross population. For each trait, the degrees of freedom (*DF*), chi-square (*Χ*^2^) statistics, and *p*-values are provided for the fixed effects of year, cross type, and the two-way interaction between year and cross type. These values indicate the significance of these factors in explaining trait variation. The suggested fixed effect structure for each trait is indicated, along with the predictive ability (correlation, "corr") of the model using the proposed fixed effects and with only the year effect (^*^). Asterisks highlight the predictive ability when year is the only fixed effect

Trait	Years	Year			Cross Type			Year ‒ Cross Type interaction			Suggested fixed effect structure	Predictive ability			
		*DF*	*Χ*^*2*^	*p*-value	*DF*	*Χ*^*2*^	*p*-value	*DF*	*Χ*^*2*^	*p*-value		corr	*p*-value	corr^*^	*p*-value
Fruit Load (0.5‒9)	2	1	261.7	< 0.001	1	2.3	0.131	1	0.7	0.404	Year	0.54	< 0.001	-	-
Fruit Weight (g)	3	2	524.4	< 0.001	1	11.4	< 0.001	2	21.7	< 0.001	Year + Cross Type + Year:Cross Type	0.38	< 0.001	0.49	< 0.001
Dry Matter (%)	3	2	1075	< 0.001	1	9.0	0.002	2	3.0	0.183	Year+Cross Type	0.30	< 0.001	0.42	< 0.001
Soluble Solids Content (°Brix)	2	1	380	< 0.001	1	7.0	0.006	1	0.1	0.731	Year+Cross Type	0.34	< 0.001	0.41	< 0.001

Model predictive ability, calculated as the correlation between predicted genomic estimated breeding values and observed mean values for each female individual, showed low to moderate accuracy across the traits. Specifically, predictive ability ranged from 0.30 for dry matter to 0.54 for fruit load. Interestingly, when only the factor year (year of season) was included in the model, predictive ability increased for all traits, indicating that the full model may be over-parameterised. These findings suggest that year is the most critical factor to consider, potentially simplifying the model without sacrificing predictive accuracy (Table 2).

Genetic parameters were estimated for four traits each considering intra- and inter-species crossing population (the *A. arguta* × *A. arguta* and *A. arguta* × *A. melanandra*). The analysis considered a full model with year of season included as a fixed effect, revealing moderate to high narrow-sense heritability (${h}_{\text{NS}}^{2}$) values of 0.41 for fruit load, 0.77 for average fruit weight, 0.52 for average dry matter, and 0.42 for ripe soluble solids content. When genetic parameters were estimated for individual years or across multiple years without including year as fixed effect, lower to moderate heritability values were observed across all traits. Overall, narrow-sense heritability was higher when the full model, including the factor year, was applied, indicating the importance of accounting for year-to-year variation in the analysis (Supplementary Table 5).

### Population effect and genomic estimated breeding value accuracy

Genomic estimated breeding value accuracy was calculated for each breeding trait of three male parents, considering different sources of population structure and the relationship structures within observation records. The breeding value accuracy of *A. arguta* 03, *A. arguta* 07, and *A. melanandra* 01 was evaluated for four traits: scored fruit load, average fruit weight, average dry matter percentage, and ripe soluble solids content. Different levels of population division were examined to understand the impact of population structure and the number of observational records (Supplementary Fig. 1).

The highest accuracy for all three male parents was observed in the total population, which included both the 2 × 13 and 13 × 3 factorial designs. Restricting the analysis to only intra-species crosses (AA × AA) resulted in high breeding value accuracy for both *A. arguta* male parents, while the accuracy for *A. melanandra* 01 significantly decreased. Conversely, when only inter-species crosses (AA × ME) were considered, accuracy for the *A. arguta* male parents dropped to levels similar to those seen for *A. melanandra* 01, which further declined.

Reducing the population size from the total to the 7 × 3 factorial subset had a minor impact on breeding value accuracy. Within this smaller group, accuracy for *A. melanandra* 01 remained lower, similar to trends seen in the total intra-species sub-population. A further decline in accuracy was noted when only the female progeny of each male parent was included, although *A. arguta* male parents consistently showed higher accuracy than *A. melanandra* 01, regardless of whether intra- or inter-species crosses were analysed. This pattern persisted across all traits, highlighting the greater reliability of breeding values for *A. arguta* male parents compared with *A. melanandra* under varying population conditions (Supplementary Fig. 1).

### Model prediction accuracy

**Fig. 3:**
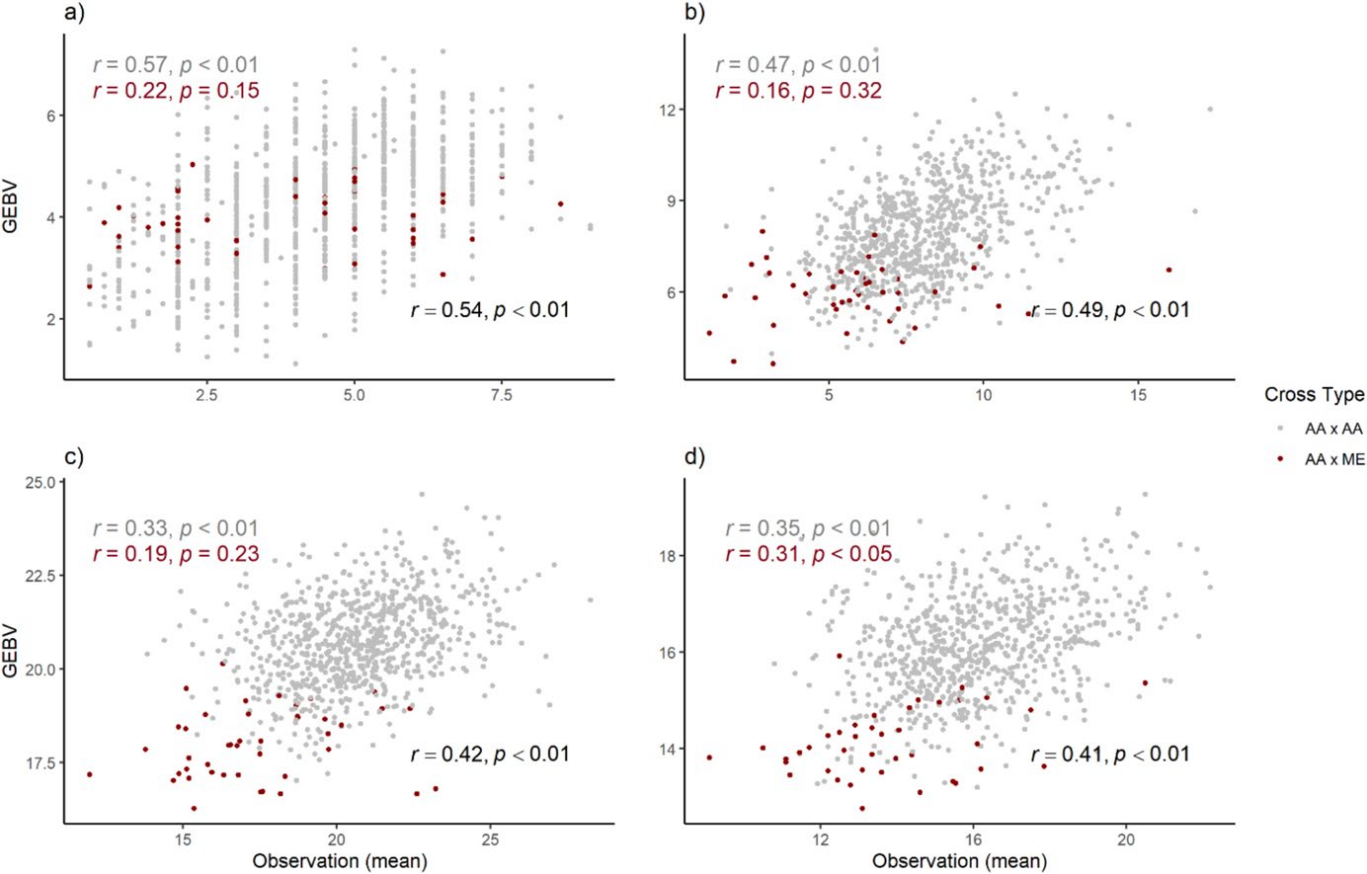
The predictive ability of four quantitative traits was assessed using leave-one-out cross-validation (LOO-CV). A linear mixed model, considering the total seedling population (*Actinidia arguta* × *A. arguta*, *A. arguta* × *A. melanandra*), was employed to calculate the correlation coefficient of genomic estimated breeding values (GEBV) with the mean observation for each trait: a) scored fruit load (0.5‒9), b) average fruit weight (in grams), c) average dry matter percentage, and d) ripe soluble solids content (in °Brix). The female progeny population was categorised by cross types, distinguishing between intra-species crosses (grey) and inter-species crosses (red). The overall (black), intra-specific (grey), and inter-specific (red) correlation coefficients (*r*) and *p*-values were calculated for each trait

Model accuracy (predictive ability) for four quantitative traits in *Actinidia* crosses was assessed using a linear mixed model through leave-one-out cross-validation, using only the year as a fixed effect. The predictive ability was assessed across the total seedling population, including both *A. arguta* × *A. arguta* (intra-species) and *A. arguta* × *A. melanandra* (inter-species) crosses. The overall predictive ability was moderate for all traits. When examining the predictive ability within specific sub-populations, the intra-species crosses showed low to moderate predictive ability with statistically significant results (*p*-value < 0.01) for all traits. However, within the inter-species crosses, predictive ability was generally low, with *p*-values exceeding 0.05, except for ripe soluble solids content, which showed a statistically significant result (*p*-value < 0.05). This indicates that predictive models perform better in intra-species crosses than in inter-species scenarios (Fig. 3).

Additionally, focused on both intra-species (AA × AA) and inter-species (AA × ME) sub-populations, a randomised selection of *A. arguta* female progeny, matching the number of individuals in the inter-species hybrids, was repeated 1,000 times to generate an average predictive ability (AA × AA^*^). The results showed that predictive ability was highest for the AA × AA sub-population across all traits, with the strongest predictive accuracy observed for fruit load (0.57) and average fruit weight (0.47), both significant at *p* < 0.001. A lower predictive ability was observed for average dry mater percentage (0.33) and ripe soluble solids content (0.35). In comparison, the predictive ability for the AA × ME sub-population was considerably lower and non-significant for all traits, indicating limited reliability in predicting breeding values for inter-species crosses. Randomised sub-sampling within the AA × AA group (AA × AA^*^) also demonstrated reduced predictive ability compared with the full intra-species population, emphasizing the influence of sample size and genetic diversity on model accuracy (Supplementary Table 6).

## Discussion

In this study, we analysed four quantitative traits within a kiwiberry breeding programme: fruit load score, average fruit weight, average dry matter, and average ripe soluble solids content. The population comprised two members of the *A. arguta*-Complex: *A. arguta* and *A. melanandra*. Intra-specific crosses of *A. arguta* and inter-specific crosses between *A. arguta* and *A. melanandra* were conducted.

All quantitative traits exhibited a continuous distribution with low to moderate skewness within intra- / inter-specific seedling populations, suggesting a cumulative effect of many genes, each contributing only a small effect. To further investigate this, we examined the homogeneity of variance for these quantitative traits between intra-specific *A. arguta* crosses and inter-specific *A. arguta* × *A. melanandra* crosses. The Levene's test results showed no significant difference in variance between the two cohorts, indicating that the variability in traits was similar for both intra- and inter-specific crosses.

Previous studies about phylogenetic relationship between the *A. arguta* and *A. melanandra* have shown high genetic similarity between both species (Huang et al. 2002; Liu et al. 2017; Bogačiovienė et al. 2019), supporting the idea of limited genetic differentiation between them. This genetic similarity was further confirmed through Principal Component Analysis (PCA) of this genotyped kiwiberry breeding population, which included two incomplete factorials. The 13 × 3 factorial analysis revealed separation of genetic variance between common male parents when PC1 and PC2 were plotted. Notably, the inter-specific genetic differences were low, as the progenies of *A. arguta* and *A. melanandra* male parents were only distinguishable by PC2 within the 13 × 3 factorial (Fig. 2), highlighting low genetic differentiation between the two species.

A linear mixed model (LMM) was employed to estimate variance components, heritability, and predictive ability for each quantitative trait, using a marker-based relationship matrix as a random effect. Our study expanded beyond primarily intra-specific crosses of *A. arguta* to include inter-specific crosses between *A. arguta* and *A. melanandra*. Given the genetic similarity between intra- and inter-species hybrids and the homogeneity of variance observed for each trait, both cross types were included in the statistical analysis.

We evaluated the impact of different fixed effects on predictive ability within the LMM. Across all traits, including the year effect as a fixed factor improved predictive ability, whereas including cross type led to a reduction in predictive ability. This suggested an over-parameterisation of the training model, making it less suitable for validation (Table 2). In the final model, which included only the year effect, we observed moderate to high heritability, consistent with a previous study focused solely on *A. arguta* genotypes (Mertten et al. 2023). In contrast, heritability was low when evaluated within each year separately or when no fixed effects were included, highlighting the importance of repeated measurements across multiple seasons (Jablonszky and Garamszegi 2024). By incorporating this factor, we ensured that the GEBV were not confounded by year-to-year environmental variability, thereby improving their reliability.

We examined further two key factors influencing predictive ability and breeding value accuracy: sample size and population structure (intra- vs. inter-specific crosses). Predictive ability was moderate within intra-specific crosses but low and not significant in inter-specific crosses. To investigate the effect of training population size, we compared predictive ability within intra-specific populations (AA × AA) and a reduced subset (AA × AA^*^), matching the sample size of the inter-specific population. Results demonstrated that training population size significantly influenced predictive ability (Supplementary Table 6). Predictive ability declined across all traits as sample size decreased, particularly within the intra-specific *A. arguta* sub-population (AA × AA) and its subset (AA × AA^*^), further emphasising the critical role of training population size in genomic prediction. In addition, predictive ability was evaluated considering the total population (AA × AA and AA × ME). Overall, the correlation between predicted breeding values and observations was low to moderate within intra-species crosses but improved for the inter-species sub-population. Nevertheless, predictive ability remained non-significant in the inter-species sub-population (Fig. 3). These findings aligned with previous studies showing a decline in predictive ability as training set size decreases (Stockwell and Peterson 2002; Wisz et al. 2008; Zhong et al. 2009; Ou and Liao 2019; Auinger et al. 2021; Wu et al. 2023).

Breeding value prediction relies on phenotypic observations of relatives, especially when own observations are unavailable. In general, parental breeding values have a high accuracy when observations from close relatives such as their progeny are considered due to the close relationship (Mertten et al. 2023). Three male parents (*A. arguta* 03, 07 and *A. melanandra* 01) were used as exemplars to investigate the effect of observation records from relatives (Supplementary Fig. 1). Population size had a less effect on breeding value accuracy than relationship, as we observed a drastic reduction of accuracy for *A. melanandra* 01 considering only observations of *A. arguta*. Similar, when only observational records of inter-species hybrids were available, a reduction of accuracy was observed for both *A. arguta* male parents.

The accuracy of breeding value prediction for parental genotypes decreased as the sample size was reduced, particularly when only one male parent was shared within the training set. This reduction in sample size not only influenced the total number of observations but also altered the population structure within the training set, thereby affecting the accuracy of breeding value predictions (Supplementary Fig. 1). Additionally, population structure was influenced by the type of hybrids involved (intra- vs. inter-species crosses). Our findings agreed with those of Lorenz and Smith (2015), who emphasised the importance of training population composition for prediction accuracy. Including closely related individuals in the training set significantly improved prediction accuracy, as accuracy for each male parent decreased when observations were limited to progeny from the other parent (Supplementary Fig. 1). This underscores that both training population structure and sample size are critical factors in achieving accurate breeding value predictions, a conclusion supported by similar studies in wheat breeding (Edwards et al. 2019; Adeyemo et al. 2020).

We acknowledge the imbalance in genotype numbers between sub-populations (AA × AA and AA × ME) and recognise that further investigations are necessary to achieve a more equitable distribution across both groups. In hybrid breeding, valuable insights can be gained from crosses involving multiple intra-species hybrids and their inter-species counterparts. To enhance breeding outcomes, careful breeding population structure must be planned, particularly in the genus *Actinidia*, where hybrid breeding is a common strategy for introducing or enhancing desirable traits (Pringle 1986; Zhong et al. 2012; Barrett et al. 2018; Nazir et al. 2024).

Since male genotypes play a crucial role in the breeding of *Actinidia* spp., using GEBV enhances the selection process by eliminating the need for progeny testing, thereby saving time and reducing costs (Datson et al. 2017; Cheng et al. 2019). Moreover, increasing the number hybrid genotypes within inter-species crosses could refine the accuracy breeding value prediction and strengthening the predictive power of genomic selection model. Nevertheless, the inclusion of inter-species hybrids in breeding value prediction requires careful analysis. Our results indicate that when different population sets were evaluated, the primary influencing factor was the genetic relationships between individuals rather than the population size. Our study provides an important foundation for exploring genomic selection within the *A. arguta* complex, highlighting the need for continued research and a more balanced representation of sub-populations to gain deeper and more comprehensive insights.

## Supplementary Information

Below is the link to the electronic supplementary material.

Supplementary file1 (DOCX 296 KB)

Supplementary file2 (DOCX 31 KB)

## Data Availability

The datasets generated during and/or analysed during the current study are available from the corresponding author on reasonable request.
