# Supplementary material for: Genomic selection in a kiwiberry breeding programme: integrating intra- and inter-specific crossing
Source: Mol Breed. 2025 Mar 7;45(3):31. doi: 10.1007/s11032-025-01550-8 (PMC11889281; doi:10.1007/s11032-025-01550-8)
Supplement: Supplementary file 1 — Supplementary file1 (DOCX 296 KB) [file 11032_2025_1550_MOESM1_ESM.docx]

**Genomic Selection in a Kiwiberry Breeding Programme: Integrating Intra- and Inter-Specific Crossing**

Molecular Breeding

Daniel Mertten*, Catherine M. McKenzie, Samantha Baldwin, Susan Thomson, Edwige J. F. Souleyre, Michael Lenhard, Paul M. Datson

***Corresponding author:**

Daniel Mertten

The New Zealand Institute for Plant and Food Research Ltd (PFR)

Auckland 1142, New Zealand

Email: Daniel.Mertten@plantandfood.co.nz


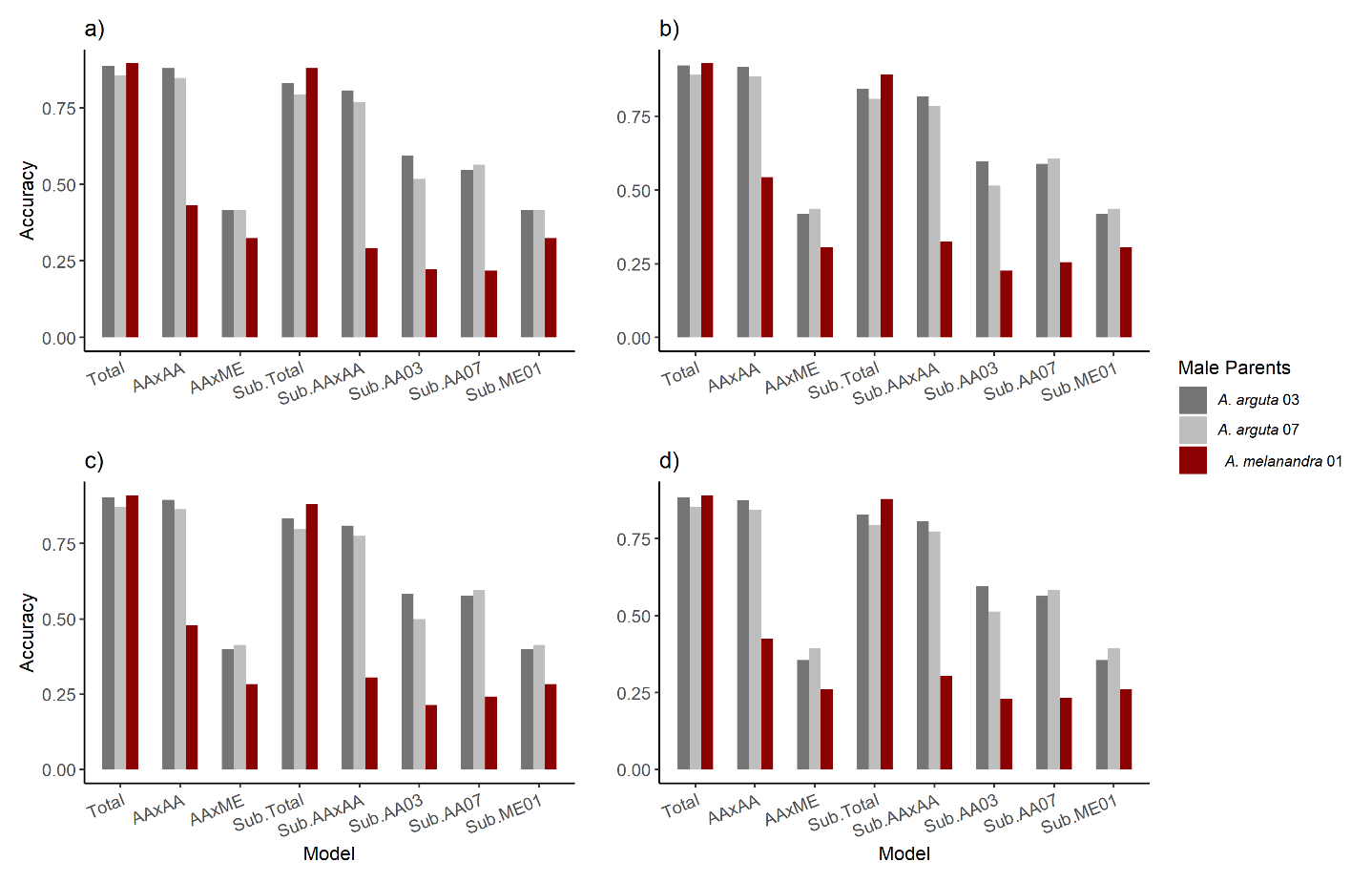


**Supplementary Fig. 1** Accuracy of male parental breeding values using different information sources. Accuracy of genomic estimated breeding values is shown for two *Actinidia arguta* male parents (*A. arguta* 03 and 07) and one *A. melanandra* male parent (*A. melanandra* 01) across four traits: (a) scored fruit load (0.5‒9), (b) average fruit weight (grams), (c) average dry matter percentage, and (d) ripe soluble solids content (°Brix). Various population groupings were evaluated, including the total population (Total), intra-species sub-population (AA×AA), inter-species sub-population (AA×ME), the total 7×3 factorial population (Sub.Total), intra-species within the 7×3 factorial (Sub.AA×AA), inter-species within the 7×3 factorial (Sub.AA×ME), and specific crosses for each male parent (Sub.AA03, Sub.AA07 and Sub.ME01)
