# Supplementary material for: Genomic selection in a kiwiberry breeding programme: integrating intra- and inter-specific crossing
Source: Mol Breed. 2025 Mar 7;45(3):31. doi: 10.1007/s11032-025-01550-8 (PMC11889281; doi:10.1007/s11032-025-01550-8)
Supplement: Supplementary file 2 — Supplementary file2 (DOCX 31 KB) [file 11032_2025_1550_MOESM2_ESM.docx]

**Genomic Selection in a Kiwiberry Breeding Programme: Integrating Intra- and Inter-Specific Crossing**

Molecular Breeding

Daniel Mertten*, Catherine M. McKenzie, Samantha Baldwin, Susan Thomson, Edwige J. F. Souleyre, Michael Lenhard, Paul M. Datson

***Corresponding author:**

Daniel Mertten

The New Zealand Institute for Plant and Food Research Ltd (PFR)

Auckland 1142, New Zealand

Email: Daniel.Mertten@plantandfood.co.nz

**Supplementary Table 1** A two-factorial breeding scheme with shared ancestry was implemented. a) 2 unrelated commercially developed female cultivars (red) of *Actinidia arguta* crossed with 13 *A. arguta* male parents (blue). b) A second factorial of 13 *A. arguta* female parents (red) crossed with *A. arguta* and *A. melanandra* male parents (blue). The second factorial design included 26 intra- and 7 inter-specific crosses

| **a)** |  | | |  | **b)** |  | | | |
| --- | --- | --- | --- | --- | --- | --- | --- | --- | --- |
|  | | **Female Parent** | |  |  | | **Male Parent** | | |
|  |  | AA-B07-01-F | AA-A01-03-F |  |  |  | *A. arguta* 03 | *A. arguta* 07 | *A. melanandra* 01 |
| **Male Parent** | AA-C01-02-M | 39 | 6 |  | **Female Parent** | AA-C01-01-F | 38 | 40 | 27 |
|  | AA-C02-01-M | 39 | 11 |  |  | - |  |  |  |
|  | AA-C02-02-M | 35 |  |  |  | - |  |  |  |
|  | AA-C03-03-M | 42 | 26 |  |  | AA-C03-01-F | 37 | 55 |  |
|  | - |  |  |  |  | AA-C03-02-F | 36 | 37 | 13 |
|  | AA-C04-02-M | 40 |  |  |  | AA-C04-01-F | 39 | 80 |  |
|  | AA-C05-02-M | 37 | 32 |  |  | AA-C05-01-F | 6 | 46 | 10 |
|  | AA-C06-01-M | 37 |  |  |  | - |  |  |  |
|  | - |  |  |  |  | AA-D01-01-F | 41 | 38 |  |
|  | - |  |  |  |  | AA-D01-02-F | 40 | 40 |  |
|  | AA-D02-02-M | 37 | 38 |  |  | AA-D02-01-F | 41 | 41 | 32 |
|  | - |  |  |  |  | AA-D04-01-F | 36 | 37 | 7 |
|  | AA-D05-01-M | 41 | 39 |  |  | - |  |  |  |
|  | AA-D06-02-M | 39 | 2 |  |  | AA-D06-01-F | 38 | 38 |  |
|  | AA-D07-02-M | 3 | 27 |  |  | AA-D07-01-F | 39 | 20 |  |
|  | AA-D07-04-M | 38 | 39 |  |  | AA-D07-03-F | 39 | 40 | 30 |
|  | AA-D07-06-M | 39 |  |  |  | AA-D07-05-F | 38 | 38 | 9 |

**Supplementary Table 2** A subset of the factorial crossing. A subset of to 13×3 factorial including seven female parents (red) crossed with three male parents (blue). The subset factorial originated from the unbalanced 13×3 factorial

|  | | **Male Parent** | | |
| --- | --- | --- | --- | --- |
|  |  | *A. arguta* 03 | *A. arguta* 07 | *A. melanandra* 01 |
| **Female Parent** | AA-C01-01-F | 38 | 40 | 27 |
|  | AA-C03-02-F | 36 | 37 | 13 |
|  | AA-C05-01-F | 6 | 46 | 10 |
|  | AA-D02-01-F | 41 | 41 | 32 |
|  | AA-D04-01-F | 36 | 37 | 7 |
|  | AA-D07-03-F | 39 | 40 | 30 |
|  | AA-D07-05-F | 38 | 38 | 9 |

**Supplementary Table 3** The complete range of recorded fruit load scores for kiwiberries

| **Score** | **No. of fruit** |
| --- | --- |
| 0 | 0 |
| 0.5 | 1–4 |
| 1 | 5–10 |
| 2 | 11–30 |
| 3 | 31–60 |
| 4 | 61–100 |
| 5 | 101–200 |
| 6 | 201–300 |
| 7 | 301–400 |
| 8 | 401–500 |
| 9 | 501+ |

**Supplementary Table 4** Quantitative traits in *Actinidia* crosses. Four quantitative traits in *Actinidia arguta* and *A. melanandra* crosses were assessed, with a variable sample size of female progeny (N). These traits were scored fruit load (0.5‒9), average fruit weight (in grams), average dry matter percentage, and an average ripe soluble solids content (in °Brix) over multiple years

| **Trait** | **N** | **Min** | **1^st^ Qu** | **Median** | **Mean** | **3^rd^ Qu** | **Max** | **Skew.** | **No. of Years** |
| --- | --- | --- | --- | --- | --- | --- | --- | --- | --- |
| **Fruit Load (0.5‒9)** | 838 | 0.5 | 3.0 | 5.0 | 4.4 | 6.0 | 9.0 | 0.1 | 2 |
| **Fruit Weight (g)** | 832 | 1.0 | 6.3 | 7.7 | 7.9 | 9.3 | 17.3 | 0.5 | 3 |
| **Dry Matter (%)** | 825 | 12.0 | 18.5 | 20.6 | 20.6 | 22.5 | 29.3 | 0.1 | 3 |
| **Soluble Solids Content (°Brix)** | 809 | 9.1 | 14.3 | 15.9 | 16.0 | 17.6 | 22.4 | 0.2 | 2 |

**Supplementary Table 5** Genetic parameters for breeding traits over single and multiple years. Genetic parameters for four breeding traits: scored fruit load (0.5‒9), average fruit weight (g), average dry matter (%), and average ripe soluble solids content (°Brix) in an *Actinidia arguta* × *Actinidia arguta* and *Actinidia arguta* × *Actinidia melanandra* cross population are shown. Data are shown separately for the first, second, and third year individually, multiple years combined without additional fixed effects, and combined multiple years, with year included as a fixed effect (^*^). For each trait, the total sample size (N) with the number of hybrids (in brackets), additive genetic variance ($\boldsymbol{\sigma}_{\boldsymbol{a}}^{\boldsymbol{2}}$), residual variance ($\boldsymbol{\sigma}_{\boldsymbol{e}}^{\boldsymbol{2}}$), and narrow-sense heritability ($\boldsymbol{h}_{\mathbf{NS}}^{\boldsymbol{2}}$) is shown

| **Trait** | **1^st^ year** | | | | **2^nd^ year** | | | | **3^rd^ year** | | | | **Multiple years** | | | | **Multiple years^*^** | | |
| --- | --- | --- | --- | --- | --- | --- | --- | --- | --- | --- | --- | --- | --- | --- | --- | --- | --- | --- | --- |
|  | **N** | $\boldsymbol{\sigma}_{\boldsymbol{a}}^{\boldsymbol{2}}$ | $\boldsymbol{\sigma}_{\boldsymbol{e}}^{\boldsymbol{2}}$ | $\boldsymbol{h}_{\mathbf{NS}}^{\boldsymbol{2}}$ | **N** | $\boldsymbol{\sigma}_{\boldsymbol{a}}^{\boldsymbol{2}}$ | $\boldsymbol{\sigma}_{\boldsymbol{e}}^{\boldsymbol{2}}$ | $\boldsymbol{h}_{\mathbf{NS}}^{\boldsymbol{2}}$ | **N** | $\boldsymbol{\sigma}_{\boldsymbol{a}}^{\boldsymbol{2}}$ | $\boldsymbol{\sigma}_{\boldsymbol{e}}^{\boldsymbol{2}}$ | $\boldsymbol{h}_{\mathbf{NS}}^{\boldsymbol{2}}$ | **N** | $\boldsymbol{\sigma}_{\boldsymbol{a}}^{\boldsymbol{2}}$ | $\boldsymbol{\sigma}_{\boldsymbol{e}}^{\boldsymbol{2}}$ | $\boldsymbol{h}_{\mathbf{NS}}^{\boldsymbol{2}}$ | $\boldsymbol{\sigma}_{\boldsymbol{a}}^{\boldsymbol{2}}$ | $\boldsymbol{\sigma}_{\boldsymbol{e}}^{\boldsymbol{2}}$ | $\boldsymbol{h}_{\mathbf{NS}}^{\boldsymbol{2}}$ |
| **Fruit Load (0.5‒9)** | 820^(42)^ | 0.89 | 2.52 | 0.26 | 714^(32)^ | 1.11 | 2.15 | 0.34 | - | | | | 838^(44)^ | 0.93 | 2.72 | 0.25 | 1.44 | 2.08 | 0.41 |
| **Fruit Weight (g)** | 676^(26)^ | 2.13 | 2.00 | 0.51 | 764^(36)^ | 2.18 | 3.69 | 0.37 | 585^(20)^ | 1.91 | 2.58 | 0.43 | 832^(43)^ | 4.09 | 2.43 | 0.63 | 5.39 | 1.64 | 0.77 |
| **Dry Matter (%)** | 612^(22)^ | 2.26 | 3.64 | 0.38 | 756^(35)^ | 2.47 | 4.01 | 0.38 | 552^(20)^ | 2.11 | 3.59 | 0.37 | 825^(43)^ | 1.95 | 5.98 | 0.25 | 3.55 | 3.23 | 0.52 |
| **Soluble Solids Content (°Brix)** | 646^(27)^ | 1.60 | 2.88 | 0.36 | 746^(35)^ | 1.62 | 3.33 | 0.33 | - | | | | 809^(40)^ | 1.29 | 4.18 | 0.24 | 2.09 | 2.86 | 0.42 |

**Supplementary Table 6** Predictive accuracy of linear mixed models in *Actinidia* crosses. Predictive ability for four quantitative traits in *Actinidia arguta* (AA) and *A. melanandra* (ME) crosses was assessed using a linear mixed model with only the year as a fixed effect. The analysis was conducted through leave-one-out cross-validation for both intra-specific (AA×AA) and inter-specific (AA×ME) sub-populations. Additionally, a randomised selection of *A. arguta* female progeny, matched to the number of individuals in the inter-species hybrids, was performed 1,000 times to calculate an average predictive ability (AA×AA^*^). The statistical significance (*p*-values) of each correlation coefficient, highlighting the model's reliability in estimating breeding values for each trait across different population structures

| **Trait** | **Predictive Ability** | | | | |
| --- | --- | --- | --- | --- | --- |
|  |  |  |  |  |  |
|  | AA×AA | *p*-value | AA×AA^*^ | AA×ME | *p*-value |
| **Fruit Load (0.5‒9)** | 0.57 | <0.001 | 0.48 | 0.09 | 0.566 |
| **Fruit Weight (g)** | 0.47 | <0.001 | 0.28 | 0.04 | 0.786 |
| **Dry Matter (%)** | 0.33 | <0.001 | 0.08 | -0.05 | 0.768 |
| **Soluble Solids Content (°Brix)** | 0.35 | <0.001 | 0.12 | 0.08 | 0.613 |
